# Economic Vulnerability among Girls at Risk for Adolescent Pregnancy: Qualitative Findings among a Clinic Sample of Girls Residing in the U.S.–Mexico Border Region

**DOI:** 10.3390/adolescents2010010

**Published:** 2022-03-02

**Authors:** Elizabeth Reed, Marissa Salazar, Alma I. Behar, Argentina E. Servin, Guadalupe X. Ayala, Jay G. Silverman, Melanie L. A. Rusch, Mari L. Zuniga, Anita Raj

**Affiliations:** 1 School of Public Health, San Diego State University, San Diego, CA 92182, USA;; 2 Center on Gender Equity and Health, Division of Global Public Health, School of Medicine, University of California San Diego, La Jolla, CA 92093, USA;; 3 Institute for Behavioral and Community Health, San Diego State University Research Foundation, San Diego, CA 92123, USA; 4 School of Social Work, San Diego State University, San Diego, CA 92182, USA;

**Keywords:** adolescent pregnancy, economic vulnerability, adolescent health, sexual health

## Abstract

**Background:**

In the U.S., research to identify the risk factors explaining high rates of adolescent pregnancy disproportionately affecting racial/ethnic minorities, including Latinas, have largely focused on social and cultural factors that influence girls’ pregnancy intentions and decisions regarding the use of contraception, as well as girls’ sexual and reproductive decision-making control in relationships. However, economic factors may play a role in increasing girls’ risk for adolescent pregnancy as well. Disproportionately high rates of adolescent pregnancy occur in areas of concentrated poverty, with higher rates among ethnic minorities. This qualitative study used a descriptive, exploratory design with a content analysis approach (1) to describe economic vulnerability in girls’ lives and (2) to identify potential ways in which economic vulnerability may influence risk factors for adolescent pregnancy among adolescent females at high risk for pregnancy recruited from a health clinic by the U.S.–Mexico border in California.

**Methods:**

Qualitative in-depth interviews were conducted among 21 girls reporting risk factors for pregnancy (including a previous pregnancy, experiences of dating violence, or having ever been diagnosed with an STI) and who were seeking sexual/reproductive health services at a clinic near the U.S.–Mexico border in California. Participants were asked about their family life, economic stressors, characteristics of intimate partner relationships, and future education/career aspirations. Interviews were analyzed using content analysis to identify common themes related to economic vulnerability and risk for pregnancy.

**Results:**

Female participants were 17 years of age on average, most (72%) were Latina, and over half (60%) were in a relationship. Participants reported a high level of economic vulnerability at home, which they felt compromised their welfare, negatively affected expectations for future educational goals, and promoted financial dependence for basic needs (e.g., food) on male partners. The latter often compromised girls’ decision-making control with relationship partners over contraceptive use and pregnancy timing.

**Conclusions:**

Study findings suggest that economic vulnerability: (a) supports economic reliance on male partners, with implications for male partner control over pregnancy decisions and (b) hinders girls’ expectations for future educational opportunities, which may reduce girls’ prioritization to delay pregnancy.

## 1. Introduction

Adolescent pregnancy and parenting disproportionately and uniquely affects adolescent girls compared to their male counterparts. In addition to girls being the ones who biologically experience pregnancy, young mothers are more likely than others to report a number of adverse social and economic challenges. Adolescent childbearing is a critical contributor to high-school dropout among girls and leads to high rates of poverty, stigma, and social isolation [1,2]. Among adolescent mothers in the U.S., only about half receive a high school diploma by age 22, compared to 90% of girls who do not give birth during adolescence [2,3]. Adolescent pregnancy also increases neonatal health risks, including prematurity and low birth weight [4].

In the U.S., while teen pregnancy rates have decreased in the past decade across the country, Latina adolescents continue to have high birth rates (31.9 births per 1000) relative to non-Latina Whites (14.3 births per 1000), with 28% of all Latinas expected to give birth prior to their 20th birthday [5,6]. Among Latina adolescent births in the U.S., 57% occur within the U.S.–Mexico border region [7]. Furthermore, Latinas of Mexican ethnicity are at highest risk for adolescent pregnancy relative to Latinas of other ethnic backgrounds [8]. In San Diego, CA, which has a large population of Latinas of Mexican origin due to its proximity to the U.S.–Mexico border, recent estimates suggest that Latinas account for three-quarters of all adolescent births [9].

In the U.S., research to identify the risk factors explaining high rates of adolescent pregnancy disproportionately affecting racial/ethnic minorities, including Latinas, have largely focused on social and cultural factors that influence girls’ pregnancy intentions and decisions regarding the use of contraception, as well as girls’ sexual and reproductive decision-making control in relationships [10,11]. Norms supportive of adolescent or early pregnancy and negative attitudes towards condom and other contraceptive use have been highlighted as important contributors to adolescent pregnancy [12,13]. Furthermore, intimate partner violence (IPV), which has been reported in up to 20% of adolescent girls, is associated with reduced sexual/reproductive autonomy (i.e., sexual and contraceptive decision-making power) among girls, and in turn, increases risk for both STI and pregnancy [14].

In addition to social and cultural factors, economic factors may play a role in increasing girls’ risk for adolescent pregnancy [15,16]. Disproportionately high rates of adolescent pregnancy occur in areas of concentrated poverty [17]. Approximately 48% of all teen mothers in the United States live below the poverty line, with higher rates among ethnic minorities [18]. While it has been well documented that adolescent pregnancy occurs in higher proportions among adolescent girls residing in low-income areas, there is a paucity of research that has explored the economic circumstances among girls in these settings that may have implications for increasing girls’ risk for adolescent pregnancy. Instead, higher rates of pregnancy among adolescent girls in low-income contexts has most often been attributed to social (e.g., early debut of sexual relationships in these contexts) or neighborhood (e.g., sexual violence) risk factors for pregnancy that are occurring disproportionately among girls residing in low-income contexts [19–21].

Among adult women, many studies have suggested that economic reliance on male partners decreases their control over sexual and reproductive decision-making power and increases risk for unintended pregnancy [22,23], yet less is known regarding the ways in which economic vulnerability may create risk for adolescent or unintended pregnancy among girls. Food insecurity, one form of economic vulnerability, has been most often associated with obesity and diabetes among young populations, particularly among adolescent girls. However, less is understood regarding how girls’ economic environment at home, including food insecurity, may increase vulnerability across other aspects of their lives, including sexual and reproductive health outcomes.

While previous research has shown that adolescent pregnancy occurs in higher proportions among girls residing in low-income neighborhoods, little is known regarding the underlying mechanisms explaining these disparities. Research among adult women suggests that economic vulnerability contributes to financial reliance on male partners, reducing women’s decision-making control over contraceptive use (including condom use), and in turn, increases unintended pregnancy [22,23]. More research is needed among adolescent girls to assess girls’ experiences of economic vulnerability, including food security, in their households and to explore possible scenarios (e.g., financial reliance on male partners, decision-making control in these relationships) that may contribute to girls’ risk for pregnancy.

Thus, the current study described qualitatively the role of economic vulnerability in girls’ lives among girls reporting risk factors for adolescent pregnancy. We conducted qualitative in-depth interviews with girls who reported risk factors for pregnancy (including a previous pregnancy, experiences of dating violence, or having ever been diagnosed with an STI) and who were seeking sexual/reproductive health services at a health clinic. The current study describes economic vulnerability in girls’ lives that girls reported during these interviews and discusses implications for pregnancy risk among this clinic sample of girls predominantly of Mexican ethnicity residing near the California U.S.–Mexico border.

## 2. Methods

This qualitative exploratory study involved conducting 21 in-depth interviews among a clinic sample of adolescent girls reporting risk factors for adolescent pregnancy. Data were analyzed using content analysis to identify common themes related to economic vulnerability and risk for pregnancy. The current qualitative study was part of a larger study aimed at assessing the risk of sexually transmitted infections (STI) and pregnancy among adolescent females residing in a neighborhood in Southeast San Diego County, near the U.S.–Mexico border [23]. This region has the highest concentration of Mexican-origin immigrants and Hispanic/Latino communities in the U.S. [24]. More than a quarter of residents in the study neighborhood live below the national federal poverty line and there are high rates of adolescent pregnancy [24].

### 2.1. Study Sample and Procedures

Participants of the larger study were recruited from a teen health clinic providing culturally and linguistically appropriate sexual and reproductive health services, including STI testing and family planning. Participation in the larger study involved the completion of a questionnaire and STI testing for Chlamydia and gonorrhea. To be eligible for the larger case-control study, individuals had to be: (a) female, (b) between the ages of 15–19 years, (c) report being sexually active in the past 6 months, and (d) willing to provide a urine sample for STI testing. Of the participants who were approached and eligible (*n* = 182), 87% (*n* = 159) participated. The most common reason for nonparticipation was lack of time. Given that girls were receiving confidential services within the clinic (e.g., parents/guardians did not have to accompany girls or be informed of their receipt of such services) and that the study involved minimal risk, parental consent was waived. In-depth interviews were conducted among a sub-set of participants (*n* = 21) who reported risk factors for pregnancy (STI history, dating violence, or previous pregnancy). Upon completing 21 interviews, no new themes emerged, indicating that we had achieved saturation. In addition to the in-depth interview data, we also used quantitative survey data to characterize the sub-sample of participants who had completed in-depth interviews.

In-depth interviews were completed either immediately after completing the survey or at a later date. We conducted in-depth interviews to understand the specific contexts and scenarios that contribute to adolescent pregnancy and STI risk among adolescent females. Trained female research assistants conducted semi-structured interviews in private rooms at the teen center. Interviews were 45–60 min, digitally recorded, transcribed, and de-identified using only a unique ID number. Upon interview completion, participants received a $20 gift card. The Human Research Protections Program at the University of California San Diego approved all study procedures.

### 2.2. Measures

Quantitative demographic data were collected to characterize the sample. Given that eligibility for being invited to complete an in-depth interview was based on reporting at least one risk factor for pregnancy, we also report the frequency of these risk factors. Risk factors for pregnancy included having ever received a diagnosis for an STI (self-reported by participants), having a history of previous pregnancy (yes/no), and having experienced physical dating violence (experienced ever being hit, pushed, shoved, hit, or slapped by a partner) or sexual dating violence (three items measuring whether participants were ever pressured, coerced, or forced to have sex).

Regarding the qualitative data from in-depth interviews, we used a semi-structured interview guide adapted for this study and based on our previous work with the population. The language used and types of probes used within the guide were piloted via focus groups prior to implementation. Participants were asked to describe general aspects of their lives, including family relationships, living situation, and relationships with male partners and friends. In addition, participants were asked about their family life, food security (e.g., where they eat the majority of their meals and access to food), characteristics of their intimate partner relationships (e.g., including decision-making, contraception use, pregnancy intentions) and future aspirations around having a family and education/career. Notably, participants were not asked directly about other experiences of economic vulnerability in their lives beyond food security; however, economic vulnerability emerged as a common theme. When asked about issues of food insecurity, participants discussed multiple economic challenges faced at home and how this affected other aspects of their lives, including relationships with males.

### 2.3. Data Analysis

An inductive, content analysis approach was used to analyze the transcripts. Content analysis involves generating and applying codes to sections of text, then reviewing the text by various codes and ‘code families’ to identify recurring themes. The coding team created an initial list of codes based on key domains and used these to code discussions. To enhance coding agreement, data were individually coded by two researchers, and coding procedures and definitions were discussed and compared for agreement. Additions of new codes or changes in code definitions were determined via consensus among the research team. Given the range of themes regarding economic vulnerability that were beyond girls’ experiences of food insecurity at home, additional codes on economic vulnerability were created to reflect these emerging themes. No new codes emerged after two-thirds of the focus groups were coded, suggesting that content saturation was achieved. For this particular analysis, we retrieved coded text regarding the economic aspects of girls’ lives, including at home (e.g., food security), in the context of relationships, and related to girls’ future aspirations. Inter-coder reliability was assessed, with 80% coder consistency or higher between coders deemed sufficient for inter-coder reliability. Interviews were coded and analyzed using Atlas Ti.

## 3. Results

### 3.1. Participant Characteristics

Participants (*n* = 21) had a mean age of 17 years (SD = 1.1) and the majority (72%) identified as Hispanic/Latina. Seventy percent of the sample reported to be White, 10% identified as Black or African American, 10% identified as Native Hawaiian/Pacific Islander, 5% as Asian, and 5% as American Indian/Alaska Native. Most participants were born in the U.S. (85%). Most participants (60%) were currently in a relationship, 10% were “dating or going out with” someone, 10% were “hooking up (i.e., having a casual relationship)”, and 10% reported being single (2 participants declined to respond to this survey item). In terms of living situation, 88% of participants lived with at least one parent, 8% lived with other adults who were not family, and 4% lived with a partner. Over half of participants (60%) reported that their parents/caregivers were stressed or highly stressed about money. Ten percent of participants had a previous history of pregnancy, almost one-third (28%) reported being diagnosed with an STI in their lifetime, and almost half experienced sexual or physical dating violence (see Table 1).

### 3.2. Qualitative Findings

Interview data highlighted that economic vulnerability was common among girls and affected multiple aspects of girls’ lives. Girls identified economic stressors at home when describing their home environment. Participants reported that economic vulnerability affected their welfare at home, and included residential instability and food insecurity. Girls also reported the financial prioritization for male family members over females within their household, further contributing to economic vulnerability among girls. Economic vulnerability was frequently noted among girls when describing challenges to achieving future educational goals. Furthermore, economic vulnerability appeared to be linked with girls’ dependence financially on male partners, which respondents reported as having implications on girls’ control over reproductive and other decision-making in these relationships. We have described each of these themes with corresponding quotes from diverse participant interviews.

#### Housing instability.

Participants reported being aware of economic vulnerability at home, and reported that it created stress and uncertainties in their lives, including residential instability. Many participants reported moving around and/or living with other family due to economic hardship in paying for housing.

My grandma passed away, so we went to Mexico for like three months and then we came back and we lost everything. We lost Section 8 [financial support for housing], we lost everything that we had. My dad was homeless during that time. It was a very hard time…witnessing my dad homeless and my grandma passing away and then … my sister leaving me and my brother for drugs. And it’s just me and my little sister, at the time I was like nine, and she was eight. So, I was like “Oh, what’s gonna happen.

[Participant was 16 years old.]

Okay, so my mom [participant lives with mom], will live with her sister to try and help with bills because rent is so high. [She] can barely pay the rent and that’s it. That’s all your money goes to unfortunately … .But I mean I don’t help with like the rent but I pay my mom’s car. I try to keep that and I pay my phone bill and my car and then I pay her life insurance so I guess I help out…

[Participant was 17 years old.]

Well … we’ve never actually like had our own house … Like my family. We’ve always … stayed with someone. Like we stayed with my—when I was like little, little, the first people that we stayed with was my aunt. And then we moved to my grandpa’s and then now we’re living at my grandma’s house. [We never lived on our own because] well my parents, they don’t have really good like credit and stuff. And then right now they’re not working.

[Participant was 16 years old.]

#### Food insecurity.

Participants also reported that lack of financial resources created challenges in access to food at home. Food insecurity occurred due to multiple circumstances, such as parents not having enough money to buy food after paying bills.

My mom used to live in hotels before we moved in together. Sometimes I would go stay with her and she just didn’t have any money [to buy food]. But she would try, she would try. I would get a bag of chips, but … [Participant reported that her mom struggled to put food on the table and sometimes all that would be available would be things like a bag of chips.]

Pay the bills or eat, or eat and you can’t pay the bills. [Participant noted that this is the message she got from her family regarding their financial situation].

We have a lot of people in our house, it cost a lot to get groceries … [At times], we don’t really have food, because we don’t have the money to go buy groceries … . [Participant noted that she and her siblings lived with parents as well as extended family.]

Participants also reported that food insecurity led them to seek meals elsewhere, such as going to friends’ houses to eat.

I eat at my friend’s house because her mom makes dinner. … I eat there … ‘cuz I never get to eat [at home].

My mom knows that I stay there [at friend’s house] to eat. [Participant reported that her mother knew that she would get food at her friend’s house, given that there was not enough food at home for her.]

#### Disproportionate allocation of limited economic resources to male siblings.

Some participants reported gender differences in how economic vulnerability affects different members of the same family. Parental preference to provide economic resources (including food) for male siblings was discussed by girls.

My little brother umm … is always with my mom, and … my mom makes sure he eats every day. … So she’s always with him, basically, like 24/7, right after he gets out of school, he’s with my mom … she really takes good care of my little brother. … He always eats, so … but that’s good for him and … it’s just something we have to go through, you know?

Oh, but I have to give these two hundred dollars to your brother. … I’m like, okay why you giving two-hundred dollars to your son when we need it. We’re still under-age, your son is thirty-two years old.

#### Reduced expectations for future education and career opportunities.

Participants reported decreased educational expectations due to experiencing economic vulnerability. Specifically, lack of financial resources was perceived to hinder participants’ future opportunities and abilities to obtain future training beyond high school.

Yeah, it’s like, not that I don’t want to go [to college], it’s that nobody’s got the money for that! Unless they start having the textbooks online for free, I don’t want to do that! [This participant stated that she was interested to go to college, but when asked more about her plans, she reported to feel that it was financially unobtainable.]

I wouldn’t have nobody to depend on. You know? I have to do it by myself. … And then just like, all the books itself for Paul Mitchell [school] are like $2000. I feel like financially … like, finances might be an issue. Like paying for college is … difficult. [This participant reported that it was her dream to go to the Paul Mitchell school; however, when asked more about it and if she saw any challenges, she described feeling that while this was her dream, it was likely unobtainable.]

One participant further explained how lack of financial resources creates multiple instabilities that could delay and/or interfere with her accomplishing educational goals, including having a family prior to accomplishing these goals.

I think boyfriend situations, my living situations, and just circumstances that come around [could prevent me from achieving my goals], … like not having a place, bouncing from place to place. And not being able to get to school or … having kids. [This participant reported having clear educational goals, but then when asked about challenges to achieve these, she also reported multiple challenges. Her response indicated she was more confident that she would be limited by multiple challenges and less confident that she would be able to achieve her goals.]

#### Economic reliance on male partners, with implications for male partner control over pregnancy.

Experiencing economic vulnerability, including food insecurity, at home led some girls to turn to male partners to provide basic needs. Participants reported that male partners often provided basic necessities, such as food and medication, which parents were unable to provide. Notably, most girls reported having older male partners, who often were no longer in school and were working.

… sometimes like my mom, she can’t afford to buy like a meal every day … he [boyfriend] asks me if I’ve eaten, and if not he’ll either take me out or invites me over to his house … [I eat] mostly with my boyfriend or when I go over to his house [boyfriend] … He buys the food.

… Even though she [mom] works like a lot, she barely has enough … so she rarely gives me money. When I get home [from school], I usually go straight to my boyfriend’s house and he usually has like food for me, or something.

[older male partner]

My mom can’t afford my medications because I take like three. He [my boyfriend] will try to get one. He’s not the richest, but he’ll try. He gets me inhalers if I need them, he provides food, like if I need hygiene products, he buys them for me.

[older male partner]

If he [older male partner] can, he sometimes gives me money he’ll send it to me through the money gram… Because he knows my situation … . He’s like, “You need clothes, and you need lady stuff. You need money to buy that! If your mom and your grandma won’t do it, someone else has to step in and do it.

[older male partner]

Financial reliance on male partners strengthened girls’ emotional connection to these partners and contributed to the partner being viewed as a someone who provides a sense of security for them similar to what parents would provide to their children.

My boyfriend is the one who … like takes care of me. … He’s helped us pay for our rent … and food. … Not only that but just like being there to care for me and love me, ‘cuz I don’t get it from my parents a lot.

He’s [older boyfriend} … very caring. He’s not a parent, but he treats me … He takes care of me. You know, he’s very husband-like. He’s very dominant. That’s how he is.

Girls also provided scenarios in which there are implications for how financial support from boyfriends may reduce girls’ decision-making power in these relationships, as well as increase their risk for IPV—and ultimately, affect their risk for pregnancy.

Like every time I’m hungry, … he [boyfriend] brings me food or every time I have to go to the store, he like picks me up and takes me, or … every time I need clothes or anything, he usually buys it for me. So umm … he’s really nice, supportive, umm … I mean he’s really jealous, but he usually gets me like whatever I want… [Boyfriend’s behavior reflective of possible IPV; participant reported he was excessively jealous and isolated her by restricting the participant’s time with friends.]

He drops me off food and even brings me like my sanitary needs (laughs) … Now he’s telling me that he wants me to get married … But I’m not, I’m not ready, I don’t wanna get married yet … I’m too young—I feel like I’m too young. But he said that if I would end up getting pregnant that we would get married.

Participant discusses her future in terms of what her boyfriend is deciding for her, despite feeling that she is too young and not ready for marriage and getting pregnant.

## 4. Discussion

The present study describes specific situations of economic vulnerability among adolescent females at high risk for pregnancy and who reside in a region near the U.S.–Mexico border in California where adolescent pregnancy is occurring in high proportions, particularly among Latina adolescents. Notably, while we did not specifically ask girls about economic vulnerability (beyond asking about food security), during conversations about their family life, girls often identified different types of financial strain at home, including residential instability of girls’ families. Girls also identified economic vulnerability as a barrier when describing their expectations for future educational and career opportunities, which has implications for girls’ risk for pregnancy given previous studies reporting low expectations for the future to be associated with girls’ de-prioritization to delay pregnancy [25–28]. Additionally, our findings indicated that economic vulnerability increases girls’ financial reliance on male partners, which has been found in previous research to be associated with male partner control over sexual/reproductive decision-making [14,29,30]. While previous research has documented the social and cultural factors that contribute to adolescent pregnancy, current study findings begin to provide context to better understand potential economic pathways that underlie disproportionately high rates of adolescent pregnancy within low-income settings or in association with economic factors at the family level (e.g., parent’s educational attainment, household income) [26,29,31]. Our work also provides insights into future research that will be needed to inform the development of interventions, including those addressing economic vulnerability among adolescents.

The present study highlights the importance of economic vulnerability in determining economic reliance on male partners, particularly older males, among adolescent girls. While gender norms are influential in males taking a lead in financial responsibilities in relationships with female partners, our study indicates economic need may also contribute to financial reliance on male partners among adolescent girls. Among Latina adolescent girls, acceptance of a male-dominant partner dynamic in the relationship may also be supported by the girl and partner’s cultural acceptance of machismo in gender roles with which the girl, partner, and family are familiar. The instances of parental preference to differentially provide food or financial support to male siblings may also reflect this acceptance in some families. Study findings are notable given that much of the previous work on economic vulnerability and financial reliance on male partners has been conducted among adult women [21,22]. Our study also provides scenarios of how economic reliance on male partners may be associated with reduced reproductive decision-making control among girls. Previous work among adult women has found that financial reliance on male partners reduces power in sexual and condom negotiations with male partners and contributes to pregnancy and IPV [14,29,30]. Studies among adults have shown that both economic dependence on male partners and IPV reduce sexual and reproductive autonomy and increase unintended pregnancy [14,32]. Our study highlights the role of economic vulnerability in financial reliance on male partners among adolescent girls, suggesting that financial reliance on male partners may be a mechanism that merits further research exploration as a possible explanatory factor in the relation between poor economic conditions and high rates of adolescent pregnancy. This factor may also play out in other poor sexual and reproductive health outcomes.

Our findings suggest that economic vulnerability may decrease girls’ perceptions of future opportunities available to them. This is an important finding given that previous research has shown that low expectations for the future may decrease intentions and behaviors (e.g., contraceptive use) to delay pregnancy. Studies have documented that among youth, low expectations for educational attainment are associated with reduced contraceptive use, de-prioritization of contraceptive use [25,26], increased sexual risk behaviors for pregnancy [27], and risk for adolescent pregnancy [28]. This previous work found that girls’ feelings of hopelessness associated with poverty were related to pregnancy attempts [33], especially when the girl is the first adolescent girl among her siblings to become pregnant [34,35], and shifted girls’ priorities away from their future training and education [25,36]. This previous work also highlighted that low future expectations is a determinant for seeking male partners who can provide economic security, which may make girls more vulnerable to IPV and reduced power over reproductive decisions [32].

Quantitative research to understand the prevalence, contexts, and consequences of economic vulnerability in adolescent girls’ lives has been limited to questions on parents’ socioeconomic status and neighborhood characteristics [31]. Our qualitative research findings may help inform the development of quantitative measures of economic vulnerability that reflect the various circumstances concerning at-risk youth, such as family dynamics, living situations, economic reliance on dating partners, future expectations, and food insecurity. Thus, our study findings related to the various scenarios where girls experience economic vulnerability may inform the development of future quantitative studies assessing associations between economic vulnerability in girls’ lives and relation to their risk for adolescent pregnancy. Quantitative assessments will be critical to inform the development of future interventions, especially those focused on reducing economic vulnerability.

## 5. Limitations

A few limitations should be noted. Our sample consisted of girls who reported risk factors for adolescent pregnancy (e.g., history of pregnancy, IPV, STI), and thus, we do not have information on whether the reported experiences with economic vulnerability are unique to girls at high-risk for pregnancy. We were not able to directly assess the relation between economic vulnerability and risk for pregnancy as it would likely be difficult for adolescent girls to make such connections. Furthermore, due to IRB concerns, we were not able to ask participants questions about family member deportation, which could be another important factor contributing to economic vulnerability in this context. We were also not able to ask girls about employment or other means of making money due to IRB concerns (with some girls under the legal working age). Our study was part of a larger study assessing STI and pregnancy risk factors; we did not directly ask girls about economic vulnerability nor how it influences their family life, expectations for the future, or relationships. Yet, through interviews with girls, the girls discussed how economic vulnerability influenced these various areas of their lives, and the findings have important implications for girls’ risk for pregnancy. While we describe specific economic challenges faced by this sample of girls at high risk for pregnancy and provide implications for how these challenges may influence risk for pregnancy, future quantitative studies will be needed to better understand these associations.

## 6. Conclusions

Our study contributes to existing research by identifying the types of economic vulnerability reported by girls, including food insecurity, family economic insecurity, and family’s prioritization of male siblings in the provision of resources. The findings highlight economic vulnerability as a concern among adolescent girls living at the U.S.–Mexico border, increasing reliance and even dependence on male partners rather than their families for supporting their basic needs. In this context, girls are made more vulnerable to male partner control over sexual and reproductive decisions. The findings also highlight how economic vulnerability may reduce girls’ expectations for future educational attainment, which has been found in previous research to reduce girls’ prioritization for delaying pregnancy. While future quantitative studies are needed, the findings suggest that economic support and empowerment for girls should be considered for inclusion in future, culturally relevant interventions. Notably, multiple research studies in other global regions outside of the U.S. have supported the effectiveness of economic interventions to improve sexual and reproductive health outcomes, including adolescent pregnancy [37,38]. By promoting financial independence, economic interventions may help girls increase future expectations and reduce financial reliance on male partners, increasing girls’ prioritization and decision-making capacity related to contraceptive use and delaying pregnancy [30,39,40]. Findings also further highlight the need for future research and interventions to consider both social and economic factors that underlie contraceptive use and reproductive health outcomes such as adolescent pregnancy.

## Figures and Tables

**Table 1.** Sample Characteristics of Adolescent Girls Participating in In-Depth Interviews (*n* = 21) Sample Characteristics *.

	Frequency (Percent)/Mean (Standard Deviation)
Age	17 (1.1)
Race	
White	15 (71.4)
Black or African America	2 (9.5)
Native Hawaiian/Pacific Islander	2 (9.5)
American Indian/Alaskan Native	1 (4.8)
Asian	1 (4.8)
Hispanic	
Yes	16 (76.2)
No	5 (23.8)
Relationship status	
In a relationship	13 (61.9)
“Dating or going out with someone”	2 (9.5)
“Hooking up”	2 (9.5)
Single (not in any relationship)	2 (9.5)
Living situation **	
Live with at least 1 parent	19 (90.5)
Live with other adults	2 (9.5)
Live with a relationship partner	1 (4.7)
Family economic stress (parents/guardians stressed or highly stressed about money)	
Yes	13 (61.9)
No	8 (38.1)
History of Pregnancy	
Yes	2 (9.5)
No	19 (90.5)
STI history (ever)	
Yes	6 (28.6)
No	15 (71.4)
Sexual or Physical Dating Violence	
Yes	10 (47.6)
No	11 (52.4)

* Some frequencies do not add up to 100% due to rounding or missing responses;

** Participants could choose more than 1 response.

## Data Availability

Data available upon request.

## References

[1] Stoner MCD; Rucinski KB; Edwards JK; Selin A; Hughes JP; Wang J; Agyei Y; Gomez-Olive FX; MacPhail C; Kahn K; The Relationship between School Dropout and Pregnancy among Adolescent Girls and Young Women in South Africa: A HPTN 068 Analysis. Health Educ. Behav 2019, 46, 559–568. 30819011 10.1177/1090198119831755 PMC6625926

[2] Hoffman SD; Maynard RA Kids Having Kids: Economic Costs & Social Consequences of Teen Pregnancy; The Urban Insitute: Washington, DC, USA, 2008.

[3] Perper K; Peterson K; Manlove J Diploma Attainment among Teen Mothers; Child Trends, Fact Sheet Publication #2010–01; Child Trends: Washington, DC, USA, 2010.

[4] Holcombe E; Peterson K; Manlove J; Scarupa HJ Ten Reasons to Still Keep the Focus on Teen Childbearing. Research Brief, Publication# 2009–2010. Child Trends 2009. Available online: https://www.childtrends.org/publications/ten-reasons-to-still-keep-the-focus-on-teen-childbearing (accessed on 22 February 2022).

[5] Mather M Trends and Challenges Facing America’s Latino Children; Population Reference Bureau: Washington, DC, USA, 2016.

[6] Ventura SJ; Hamilton BE; Matthews TJ National and State Patterns of Teen Births in the United States, 1940–2013. Div. Vital Stat. Natl. Vital Stat 2014, 63, 1–34. 25142408

[7] McDonald JA; Mojarro O; Sutton PD; Ventura SJ Adolescent Births in the Border Region: A Descriptive Analysis Based on US Hispanic and Mexican Birth Certificates. Matern. Child Health J 2015, 19, 128–135. 24820518 10.1007/s10995-014-1503-2

[8] Martin JA; Hamilton BE; Osterman MJ; Driscoll AK Births: Final Data for 2019; National Center for Health Statistics: Hyattsville, MD, USA, 2021. Available online: https://www.cdc.gov/nchs/data/nvsr/nvsr70/nvsr70-02-508.pdf (accessed on 22 February 2022). 33814033

[9] Martin JA; Hamilton BE; Osterman MJ; Driscoll AK; Drake P Births: Final Data for 2016; National Center for Health Statistics: Hyattsville, MD, USA, 2018. Available online: https://www.cdc.gov/nchs/data/nvsr/nvsr67/nvsr67_01.pdf (accessed on 22 February 2022). 29775434

[10] Killoren S; Zeiders E; Updegraff K; Umaña-Taylor H The Sociocultural Context of Mexican-Origin Pregnant Adolescents’ Attitudes toward Teen Pregnancy and Links to Future Outcomes. J. Youth Adolesc 2016, 45, 887–899. 26573862 10.1007/s10964-015-0387-9 PMC4826852

[11] Sommer P; Kelley M; Norr K; Patil C; Vonderheid S Mexican American Adolescent Mothers’ Lived Experience: Grounded Ethnicity and Authentic Mothering. Glob. Qual. Nurs. Res 2019, 6, 1–15. 10.1177/2333393619850775 PMC6539571 31192272

[12] Aparicio EM; Vanidestine T; Zhou K; Pecukonis EV Teenage Pregnancy in Latino Communities: Young Adult Experiences and Perspectives of Sociocultural Factors. Fam. Soc 2016, 97, 50–57.

[13] Kirby D Antecedents of adolescent initiation of sex, contraceptive use, and pregnancy. Am. J. Health Behav 2002, 26, 473–485. 12437022 10.5993/ajhb.26.6.8

[14] Miller E; Decker MR; Reed E; Raj A; Hathaway JE; Silverman JG Male partner pregnancy-promoting behaviors and adolescent partner violence: Findings from a qualitative study with adolescent females. Ambul. Pediatr 2007, 7, 360–366. 17870644 10.1016/j.ambp.2007.05.007

[15] Harling G; Subramanian SV; Bärnighausen T; Kawachi I Socioeconomic disparities in sexually transmitted infections among young adults in the United States: Examining the interaction between income and race/ethnicity. Sex. Transm. Dis 2013, 40, 575. 23965773 10.1097/OLQ.0b013e31829529cf PMC3752095

[16] Haar K; Bremer V; Houareau C; Meyer T; Desai S; Thamm M; Hamouda O Risk factors for Chlamydia trachomatis infection in adolescents: Results from a representative population based survey in Germany, 2003–2006. Eurosurveillance 2013, 18, 20562. 23987832 10.2807/1560-7917.es2013.18.34.20562

[17] United States Census Bureau. Quick Facts-San Diego County, California-Population Estimates. 2014. Available online: https://www.census.gov/quickfacts/fact/table/sandiegocountycalifornia/PST045221 (accessed on 22 February 2022).

[18] Kaye K Why It Matters: Teen Childbearing and Infant Health. The National Campaign to Prevent Teen and Unplanned Pregnancy. 2014. Available online: https://www.courts.ca.gov/documents/BTB24-2J-11.pdf (accessed on 22 February 2022).

[19] Dupéré V; LaCourse É; Willms JD; Leventhal T; Tremblay RE Neighborhood Poverty and Early Transition to Sexual Activity in Young Adolescents: A Developmental Ecological Approach. Child Dev 2008, 79, 1463–1476. 18826536 10.1111/j.1467-8624.2008.01199.x

[20] Roche K; Leventhal T Beyond Neighborhood Poverty: Family Management, Neighborhood Disorder, and Adolescents’ Early Sexual Onset. J. Fam. Psychol 2009, 23, 819–827. 20001140 10.1037/a0016554

[21] Reed E; Salazar M; Behar AI; Agah N; Silverman JG; Minnis AM; Rusch ML; Raj A Cyber sexual harassment: Prevalence and association with substance use, STI, and poor mental health outcomes among adolescent girls in the US. J. Adolesc 2019, 75, 53–62. 31344557 10.1016/j.adolescence.2019.07.005 PMC6716784

[22] Babcock RL; Deprince AP Factors contributing to ongoing intimate partner abuse: Childhood betrayal trauma and dependence on one’s perpetrator. J. Interpers. Violence 2013, 28, 1385–1402. 23266993 10.1177/0886260512468248

[23] Dhungel S; Dhungel P; Dhital SR; Stock C Is economic dependence on the husband a risk factor for intimate partner violence against female factory workers in Nepal? BMC Women Health 2017, 17, 82. 10.1186/s12905-017-0441-8 PMC5598058 28903741

[24] Carmen DW; Proctor BD; Smith JC Current Population Reports, P60–235, Income, Poverty, and Health Insurance Coverage in the United States, US Census Bureau 2007. 2008. Available online: https://www.census.gov/library/publications/2008/demo/p60-235.html (accessed on 22 February 2022).

[25] Minnis AM; Marchi K; Ralph L; Biggs MA; Combellick S; Arons A; Brindis CD; Braveman P Limited socioeconomic opportunities and Latina teen childbearing: A qualitative study of family and structural factors affecting future expectations. J. Immigr. Minority Health 2013, 15, 334–340. 10.1007/s10903-012-9653-z PMC3479330 22678305

[26] Oman RF; Vesely SK; Aspy CB; Tolma EL; Gavin L; Bensyl DM; Mueller T; Fluhr JD A longitudinal study of youth assets, neighborhood conditions, and youth sexual behaviors. J. Adolesc. Health 2013, 52, 779–785. 23402985 10.1016/j.jadohealth.2012.12.005

[27] Childs GD; Knight C; White R Never-Pregnant African American Adolescent Girls’ Perceptions of Adolescent Pregnancy. J. Pediatr. Nurs 2015, 30, 310–320. 25236337 10.1016/j.pedn.2014.08.012 PMC4362966

[28] Harden A; Brunton G; Fletcher A; Oakley A Teenage pregnancy and social disadvantage: Systematic review integrating controlled trials and qualitative studies. BMJ 2009, 339, b4254. 19910400 10.1136/bmj.b4254 PMC2776931

[29] Penman-Aguilar A; Carter M; Snead MC; Kourtis A Socioeconomic disadvantage as a social determinant of teen childbearing in the US. Public Health Rep 2013, 128 (Suppl. 1), 5–22. 10.1177/00333549131282S102 PMC3562742 23450881

[30] Biello KB; Sipsma HL; Ickovics JR; Kershaw T Economic dependence and unprotected sex: The role of sexual assertiveness among young urban mothers. J. Urban Health 2010, 87, 416–425. 20352355 10.1007/s11524-010-9449-1 PMC2871080

[31] Maness SB; Buhi ER Associations between social determinants of health and pregnancy among young people: A systematic review of research published during the past 25 years. Public Health Rep 2016, 131, 86–99. 26843674 10.1177/003335491613100115 PMC4716476

[32] Al Riyami A; Afifi M; Mabry RM Women’s autonomy, education and employment in Oman and their influence on contraceptive use. Reprod. Health Matters 2004, 12, 144–154. 15242223 10.1016/s0968-8080(04)23113-5

[33] Fedorowicz AR; Hellerstedt WL; Schreiner PJ; Bolland JM Associations of adolescent hopelessness and self-worth with pregnancy attempts and pregnancy desire. Am. J. Public Health 2014, 104, e133–e140. 24922147 10.2105/AJPH.2014.301914 PMC4103254

[34] East PL; Chien NC Family dynamics across pregnant Latina adolescents’ transition to parenthood. J. Fam. Psychol 2010, 24, 709–720. 21171769 10.1037/a0021688 PMC3662293

[35] East PL; Slonim A; Horn EJ; Trinh C; Reyes BT How an adolescent’s childbearing affects siblings’ pregnancy risk: A qualitative study of Mexican American youths. Perspect. Sex. Reprod. Health 2009, 41, 210–217. 20444174 10.1363/4121009 PMC3711254

[36] So S; Voisin DR; Burnside A; Gaylord-Harden NK Future orientation and health related factors among African American adolescents. Child. Youth Serv. Rev 2016, 61, 15–21.

[37] Bearinger LH; Sieving RE; Ferguson J; Sharma V Global perspectives on the sexual and reproductive health of adolescents: Patterns, prevention, and potential. Lancet 2007, 369, 1220–1231. 17416266 10.1016/S0140-6736(07)60367-5

[38] Chandra-Mouli V; Svanemyr J; Amin A; Fogstad H; Say L; Girard F; Temmerman M Twenty years after International Conference on Population and Development: Where are we with adolescent sexual and reproductive health and rights? J. Adolesc. Health 2015, 56, S1–S6. 10.1016/j.jadohealth.2014.09.015 25528975

[39] Do M; Kurimoto N Women’s empowerment and choice of contraceptive methods in selected African countries. Int. Perspect. Sex. Reprod. Health 2012, 38, 23–33. 22481146 10.1363/3802312

[40] Postmus JL; Plummer S-B; McMahon S; Murshid NS; Kim MS Understanding economic abuse in the lives of survivors. J. Interpers. Violence 2012, 27, 411–430. 21987509 10.1177/0886260511421669

